# The Energy Sensor AMPKα1 Is Critical in Rapamycin-Inhibition of mTORC1-S6K-Induced T-cell Memory

**DOI:** 10.3390/ijms23010037

**Published:** 2021-12-21

**Authors:** Anjuman Ara, Aizhang Xu, Khawaja Ashfaque Ahmed, Scot C. Leary, Md. Fahmid Islam, Zhaojia Wu, Rajni Chibbar, Jim Xiang

**Affiliations:** 1 Cancer Research Cluster, Saskatchewan Cancer Agency, 20 Campus Drive, Saskatoon, SK S7N 4H4, Canada; ana277@mail.usask.ca (A.A.); aix705@mail.usask.ca (A.X.); mf.islam@usask.ca (M.F.I.); zhaojia.wu@usask.ca (Z.W.); 2 Division of Oncology, College of Medicine, University of Saskatchewan, 107 Wiggins Road, Saskatoon, SK S7N 5E5, Canada; 3 Department of Pathology, Western College of Veterinary Medicine, University of Saskatchewan, Saskatoon, SK S7N 5B4, Canada; kaa201@mail.usask.ca; 4 Department of Biochemistry, Microbiology and Immunology, College of Medicine, University of Saskatchewan, 107 Wiggins Road, Saskatoon, SK S7N 5E5, Canada; scot.leary@usask.ca; 5 Department of Pathology, College of Medicine, University of Saskatchewan, 107 Wiggins Road, Saskatoon, SK S7N 5E5, Canada; Rajni.Chibbar@saskhealthauthority.ca

**Keywords:** rapamycin, mTORC1, S6K, AMPKα1, T-cell memory, FOXO1, autophagy, ULK1, mitochondrial biogenesis, fatty acid oxidation, glycolysis

## Abstract

Energy sensors mTORC1 and AMPKα1 regulate T-cell metabolism and differentiation, while rapamycin (Rapa)-inhibition of mTORC1 (RIM) promotes T-cell memory. However, the underlying pathway and the role of AMPKα1 in Rapa-induced T-cell memory remain elusive. Using genetic and pharmaceutical tools, we demonstrate that Rapa promotes T-cell memory in mice in vivo post *Listeria monocytogenesis* rLmOVA infection and in vitro transition of effector T (T_E_) to memory T (T_M_) cells. IL-2- and IL-2+Rapa-stimulated T [IL-2/T and IL-2(Rapa+)/T] cells, when transferred into mice, differentiate into short-term IL-7R^−^CD62L^−^KLRG1^+^ T_E_ and long-lived IL-7R^+^CD62L^+^KLRG1^−^ T_M_ cells, respectively. To assess the underlying pathways, we performed Western blotting, confocal microscopy and Seahorse-assay analyses using IL-2/T and IL-2(Rapa+)/T-cells. We determined that IL-2(Rapa+)/T-cells activate transcription FOXO1, TCF1 and Eomes and metabolic pAMPKα1(T_172_), pULK1(S_555_) and ATG7 molecules and promote mitochondrial biogenesis and fatty-acid oxidation (FAO). We found that rapamycin-treated AMPKα-deficient AMPKα1-KO IL-2(Rapa+)/T_M_ cells up-regulate transcription factor HIF-1α and induce a metabolic switch from FAO to glycolysis. Interestingly, despite the rapamycin treatment, AMPKα-deficient T_M_ cells lost their cell survival capacity. Taken together, our data indicate that rapamycin promotes T-cell memory via transcriptional FOXO1-TCF1-Eomes programs and AMPKα1-ULK1-ATG7 metabolic axis, and that AMPKα1 plays a critical role in RIM-induced T-cell memory.

## 1. Introduction

During an acute infection, antigen-specific CD8^+^ cytotoxic T lymphocytes (CTLs) activated by antigen-presenting cells rapidly proliferate and differentiate into effector T (T_E_) cells [1]. These T_E_ cells constitute an important arm of adaptive immunity and provide protection against pathogens [2]. After pathogen clearance, the majority (90–95%) of these T_E_ cells are eliminated by apoptosis during the contraction phase; however, a small fraction (5–10%) survive and further differentiate into CD8^+^ memory T (T_M_) cells. These CD8^+^ T_M_ cells respond robustly upon reencountering the same antigen and result in a recall response that efficiently prevents pathogen-induced re-infection [2]. Therefore, a greater understanding of the mechanisms of T-cell survival and memory formation is critical for developing vaccine and immunotherapeutic strategies against cancer and infectious diseases [3].

Growing evidence indicates T-cell metabolism is an important factor in T-cell differentiation and immunity [4,5,6]. During T-cell activation and differentiation, cellular metabolism undergoes dynamic changes [4,5,6]. Naive T-cells exhibit basal levels of nutrient uptake and mainly use mitochondrial oxidative phosphorylation (OXPHOS) and fatty acid oxidation (FAO) for energy production [4,5,6]. Upon activation, most T-cells meet the energetic demands of proliferation and performing their effector functions as short-term T_E_ cells by switching from catabolic FAO to aerobic glycolysis [4,5,6]. Fuel preference is subsequently altered in a small subpopulation of these T-cells, gradually resetting back to a more catabolic FAO state to support T-cell memory formation in the memory phase [7,8]. However, the underlying mechanism is not fully understood.

CD8^+^ T_M_ cells have two unique characteristics; namely, expression of the T_M_ cell phenotype (CCR7^+^CD62L^+^IL-7R^+^) and the capacity for recall responses upon secondary stimuli [9,10]. The T_M_ cell phenotype plays an important role in the survival and homeostasis of these cells. For example, the chemokine receptor CCR7 and adhesion molecule CD62L control T_M_ cell migration and homing to lymphoid organs, where CCR7^+^CD62L^+^IL-7R^+^ CD8^+^ T_M_ cells are capable of inducing robust recall responses when supported by stromal cell-derived IL-7 [10,11,12]. The transcriptional factor FOXO1 (forkhead box protein-O1) and its two down-stream targets TCF1 (T-cell factor-1) and Eomes (eomesodermin) regulate the T_M_ cell phenotype and differentiation [13,14,15,16,17].

It is well established that mammalian target of rapamycin complex-1 (mTORC1) and adenosine monophosphate-activated protein kinase alpha-1 (AMPKα1) are two evolutionally conserved energy sensors that regulate cellular metabolism and differentiation [18,19]. mTORC1 modulates various cellular processes including T-cell metabolism, proliferation, migration, survival, and differentiation by regulating the activity of downstream targets ribosomal S6K (S6 kinase) and eIF4E (eukaryotic translation initiation factor-4E) [2,20]. S6K in turn regulates its substrate ribosomal S6, which is routinely used as a proxy of mTORC1 activity. Ahmed’s group originally reported in 2009 that rapamycin (Rapa)-inhibition of mTORC1 (RIM) promoted CD8^+^ T-cell memory [21]. More and more evidence has since been generated to support this finding, showing that RIM maintains T-cell plasticity and promotes antigen-specific CD8^+^ central memory in T-cells [22,23,24]. However, the transcriptional and metabolic pathways by which RIM promotes long-term CD8^+^ T-cell memory are not well established.

AMPKα1 regulates catabolic processes by activating the essential autophagy kinase Unc-51-like autophagy-activating kinase-1 (ULK1), and stimulating mitochondrial biogenesis which leads to preferential use of FAO metabolism for energy production [25]. Autophagy is a cytosolic self-recycling process in which proteins and organelles are degraded via lysosomes to provide essential metabolic intermediates to cells [26,27]. Two major components of the autophagy pathway are ULK1 and autophagy-related gene-7 (ATG7) [28]. Phosphorylation of AMPKα1 (pAMPKα1, T_172_) leads to activation of ULK1 and ATG7, which in turn promote autophagy [29] and mitochondrial biogenesis as well as FAO metabolism [25]. The energy sensor AMPKα1 has also been found to regulate T-cell memory [30,31]. However, its critical role in RIM-induced T-cell memory formation is elusive.

In this study, we sought to elucidate the molecular mechanism by which RIM induces T-cell memory formation by applying state-of-the-art genetic and pharmaceutical tools in various in vivo and in vitro experiments. To assess Rapa-promoted T-cell memory, we conducted kinetic flow cytometry analysis in C57BL/6 (B6) mice infected with recombinant *Listeria monocytogenes* rLmOVA to demonstrate that in vivo treatment with Rapa promotes long-term T-cell survival and IL-7R^+^CD62L^+^KLRG1^−^ T_M_ cell formation. We also prepared in vitro IL-2- and IL-2+Rapa-stimulated T [IL-2/T and IL-2(Rapa+)/T] cells for further systematic characterization. We show that after being adoptively transferred into B6.1 mice, IL-2/T and IL-2(Rapa+)/T-cells display high and low levels of mTORC1-S6K signaling and become short-lived IL-7R^−^CD62L^−^KLRG1^+^ T_E_ cells and long-lived IL-7R^+^CD62L^+^KLRG1^−^ T_M_ cells, respectively. We also demonstrate that in vitro Rapa-induced IL-2(Rapa+)/T_M_ cells up-regulate CD45RA, a stem cell-like memory T (T_SCM_) cell marker [32]. To elucidate molecular pathways by which Rapa-induced IL-2(Rapa+)/T_M_ cell formation, we performed Western blot, confocal and electron microscopy and Seahorse assay analyses using IL-2/T and IL-2(Rapa+)/T-cells. We establish that IL-2(Rapa+)/T_M_ cells activate the FOXO1-TCF1-Eomes transcriptional programs controlling T_M_-cell differentiation and upregulate the AMPKα1-ULK1-ATG7 metabolic axis regulating mitochondrial biogenesis and FAO metabolism. To assess the critical role of the energy sensor AMPKα1 in RIM-induced T-cell memory, we genetically engineered AMPKα1 knock-out (KO)/OTI mice and isolated AMPKα1 KO IL-2(Rapa+)/T_M_ cells from these animals. We show that in vitro the AMPKα1 deficiency up-regulates the expression of the transcription factor hypoxia-inducible factor-1 (HIF-1α) and induces a metabolic switch from FAO to glycolytic metabolism. These AMPKα1 KO IL-2(Rapa+)/T_M_ cells also lose long-term survival upon their post-adoptive transfer into B6.1 mice.

## 2. Results

### 2.1. Rapamycin Promotes T-cell Survival and Memory Formation In Vivo Post Infection with Recombinant Listeria Monocytogenes rLmOVA

**Figure 1:**
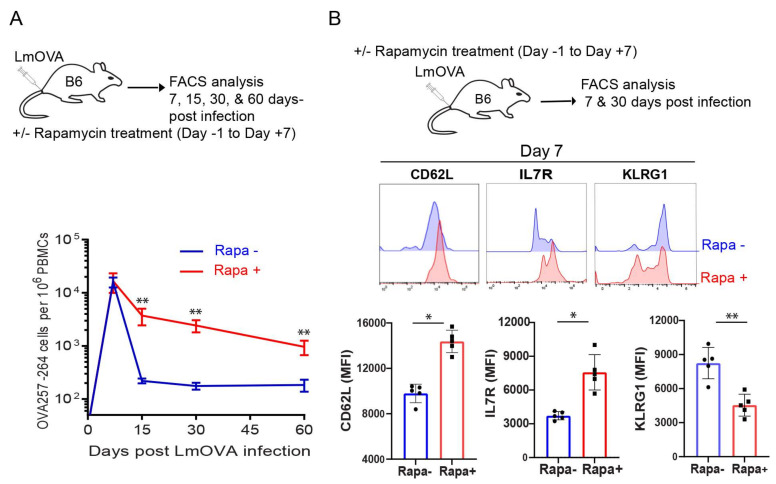
Rapamycin promotes in vivo CD8^+^ T-cell survival and memory formation. (**A**) Schematic diagram of rLmOVA infection in C57BL/6 mice. Mice (*n* = 5/group) were intravenously injected on day 0 with 2000 CFUs of rLmOVA. Mice were treated with rapamycin from day −1 to day +7. Rapamycin not treated mice received saline and served as controls. Longitudinally tracked kinetics of OVA-specific CD8^+^ T-cells in blood using flow cytometry. The line graph shows the frequencies of OVA-specific CD8^+^ T-cells in rapamycin-treated (red) and not treated (blue) groups. (**B**) Cell phenotype of the OVA-specific CD8^+^ T-cells in the peripheral blood was analyzed using cell surface memory markers by flow cytometry. Data presented as means ± SEM are representative of two independent experiments with similar results (*n* = 2–3/group/experiment). * *p* < 0.05, ** *p* < 0.01 by two-tailed Student’s *t*-test.

To confirm that Rapa promotes T-cell memory, B6 mice were infected with recombinant *Listeria monocytogenes* rLmOVA and treated with rapamycin daily (days -1 to 7 post-infection). OVA-specific CD8^+^ T-cell responses were measured by flow cytometry analysis of mouse peripheral blood stained with PE-labelled H-2K^b^/OVA_SIINFEKL_ (OVAI) peptide (PE-tetramer) and FITC-labelled anti-CD8^+^ antibody (FITC-CD8) at 7, 15, 30 and 60 d post infection. We found a similar frequency of OVA-specific CD8^+^ T-cells in mice treated with or without Rapa at the peak of CD8^+^ T-cell responses 7 days post-infection, but a decreased contraction of the CD8^+^ T-cell response in the Rapa-treated group when compared to the untreated group (Figure 1A). In fact, the higher frequency of CD8^+^ T-cells was maintained at days 30 and 60, when CD8^+^ T-cells differentiated into T_M_ cells (Figure 1A). At the peak of CD8^+^ T-cell responses, T_E_ cells consist of two subsets: IL-7R^−^CD62L^−^KLRG1^+^ short-lived effector cells (SLECs) and IL-7R^+^CD62L^−^KLRG1^−^ memory precursor effector-cells (MPECs). The SLECs are terminally differentiated and poised for apoptosis while the MPECs show a greater propensity to survive and differentiate into T_M_ cells [33,34]. Therefore, we next examined the phenotype of CD8^+^ T-cells in infected mice by gating PE-tetramer^+^CD8^+^ T-cells 7 days post-infection for further measurement of the above three markers. We demonstrated that CD8^+^ T-cells in Rapa-treated mice expressed more of the T_M_ cell markers IL-7R and CD62L, but less of the T_E_ cell marker KLRG1, whereas the reciprocal expression pattern of these cell surface markers was observed in CD8^+^ T-cells derived from untreated mice (Figure 1B). These data indicate that Rapa treatment induces CD8^+^ T-cells to differentiate into MPECs, leading to more T-cell memory formation post-infection with recombinant *Listeria monocytogenes* rLmOVA (Figure 1A).

### 2.2. Rapamycin Promotes the Transition of T_E_ into Long-Term CD45RA^+^ Stem Cell-Like T_M_ Cells In Vitro

**Figure 2:**
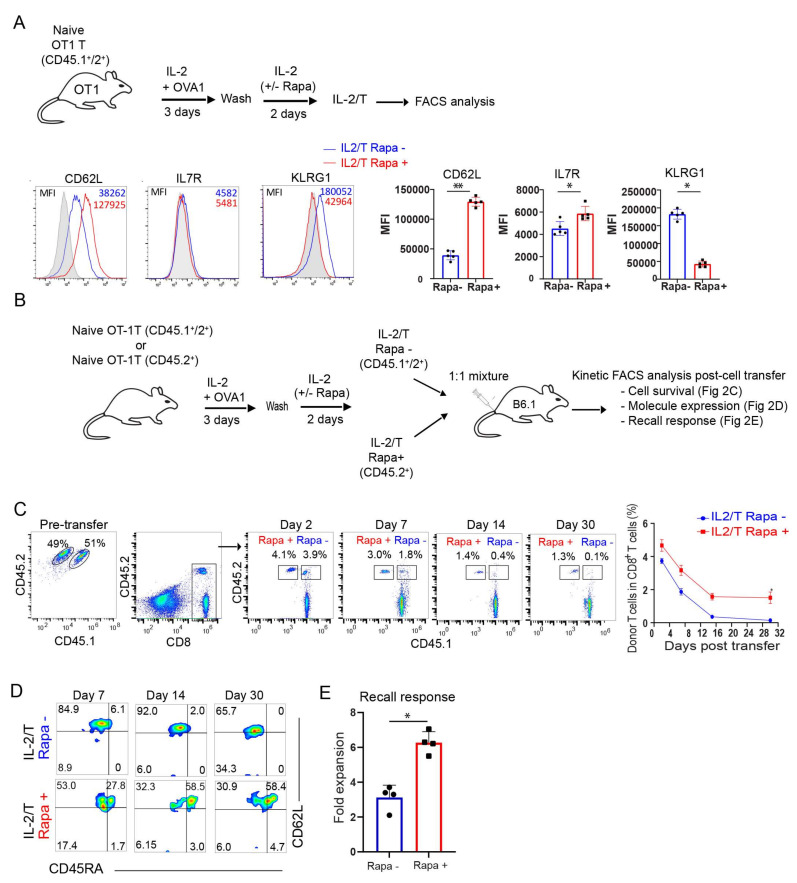
Rapamycin promotes in vitro transition of T_E_ into long-term CD45RA^+^ stem cell-like T_M_ cells. (**A**) Schematic diagram indicates splenic CD8^+^ T-cell culture using OT-1 mice (OVA-specific CD8^+^ T-cells) to generate rapamycin-treated or not treated effector T-cells. The OVA-specific CD8^+^ T-cells were activated by 0.10 nM OVAI-peptide and cultured for 3 days in IL-2 containing media. Following the wash, activated T-cells were cultured in the presence or absence of rapamycin (100 nM) for 2 additional days in IL-2 containing media. The histogram indicates representative data for the expression of cell surface T-cell markers. The line graphs (*n* = 5) show the expression of cell surface T-cell markers. (**B**) Congenic OT-1 mice from CD45.2^+^ or CD45.1^+^/2^+^ background were used to generate OVA-specific CD8^+^ T effector cells in the presence or absence of rapamycin. Rapamycin treated (CD45.2^+^) or not treated (CD45.1^+^/2^+^) effector T-cells were mixed 1:1 and adoptively transferred into CD45.1 mice. (**C**) The cell survival kinetics of adoptively transferred rapamycin-treated (CD45.2^+^) or not treated (CD45.1^+^/2^+^) effector T-cells in total host CD8+ T-cells were analyzed longitudinally in peripheral blood by flow cytometry. Flow panels (left) and line graph (right) show the relative percentage of rapamycin-treated (CD45.2^+^) or not treated (CD45.1^+^/2^+^) effector T-cells post-adoptive transfer (*n* = 4). (**D**) Flow panels indicate cell phenotype of rapamycin-treated (CD45.2^+^) or not treated (CD45.1^+^/2^+^) effector T-cells post-adoptive transfer (*n* = 4). (**E**) Graph shows recall response of rapamycin-treated (CD45.2^+^) or not treated (CD45.1^+^/2^+^) T-cells 30 days after the adoptive transfer and 5 days post-rLmOVA (5000 CFU) intravenous challenge (*n* = 4). Data presented as means ± SEM are representative of two independent experiments with similar results (*n* = 2–3/group/experiment). * *p* < 0.05, ** *p* < 0.01 by two-tailed Student’s *t*-test.

Rapa is reportedly capable of promoting the transition of T_E_ into T_M_ cells in vivo [21]. Based upon this capability, we developed an in vitro culture approach using Rapa treatment to promote the transition of T_E_ into T_M_ cell precursors, similar to past protocols that have used the pro-survival cytokines IL-7 and IL-15 for the same purpose [35,36,37]. Our in vitro culture-prepared T_E_ and T_M_ cells approximate the T_E_ and T_M_ cell differentiation program observed in vivo. Briefly, naïve OTI CD8^+^ T-cells were cultured in a medium containing OVAI peptide and IL-2 for 3 d, and then these active CD8^+^ T-cells were cultured for another 2 d in medium containing IL-2 in the absence or presence of Rapa to generate IL-2/T and IL-2(Rapa+)/T-cells, respectively (Figure 2A). Subsequent flow cytometry analysis found that IL-2(Rapa+)/T-cells up-regulated IL-7R and CD62L and down-regulated KLRG1, while the reciprocal response was observed for IL-2/T-cells (Figure 2A). To measure survival of these T-cell subsets in vivo, we adoptively transferred equal amounts of in vitro-prepared CD45.1^+^/2^+^ IL-2/T and CD45.2^+^ IL-2(Rapa+)/T-cells into CD45.1^+^ B6.1 mice (Figure 2B). This approach allows us to separately analyze the survival of IL-2/T and IL-2(Rapa+)/T-cells in B6.1 mouse peripheral blood by kinetic flow cytometry analyses post-cell transfer (Figure 2C), as previously described [36]. We found significantly more IL-2(Rapa+)/T than IL-2/T donor cells in host mice at different time points post-cell transfer. At 30 days post-cell transfer, IL-2(Rapa+)/T-cells were 13-fold more abundant than IL-2/T donor cells in host mice, indicating IL-2(Rapa+)/T-cells survive much longer than IL-2/T-cells. We then characterized these T-cells by measuring expression of the T_M_ cell marker CD62L and the T_SCM_ cell marker CD45RA [32]. Interestingly, we found that all IL-2/T and IL-2(Rapa+)/T-cells expressed CD62L while 58% of the IL-2(Rapa+)/T but none of the IL-2/T-cells expressed CD45RA at days 14 and 30 post-cell transfer (Figure 2D), indicating Rapa promotes CD45RA^+^ T_SCM_ cells. To assess their functionality, we performed T_M_ cell recall responses by infecting T-cell-transferred mice 30 d post-cell transfer with rLmOVA. Flow cytometry analysis of mouse peripheral blood 4 d post-infection showed that T_M_ cells derived from IL-2(Rapa+)/T-cells exhibited roughly a 2-fold greater expansion than T_M_ cells derived from IL-2/T-cells (Figure 2E).

### 2.3. IL-2(Rapa+)/T-cells Suppress mTORC1/S6K Signaling and Activate the FOXO1-TCF1-Eomes Transcriptional Pathway

**Figure 3:**
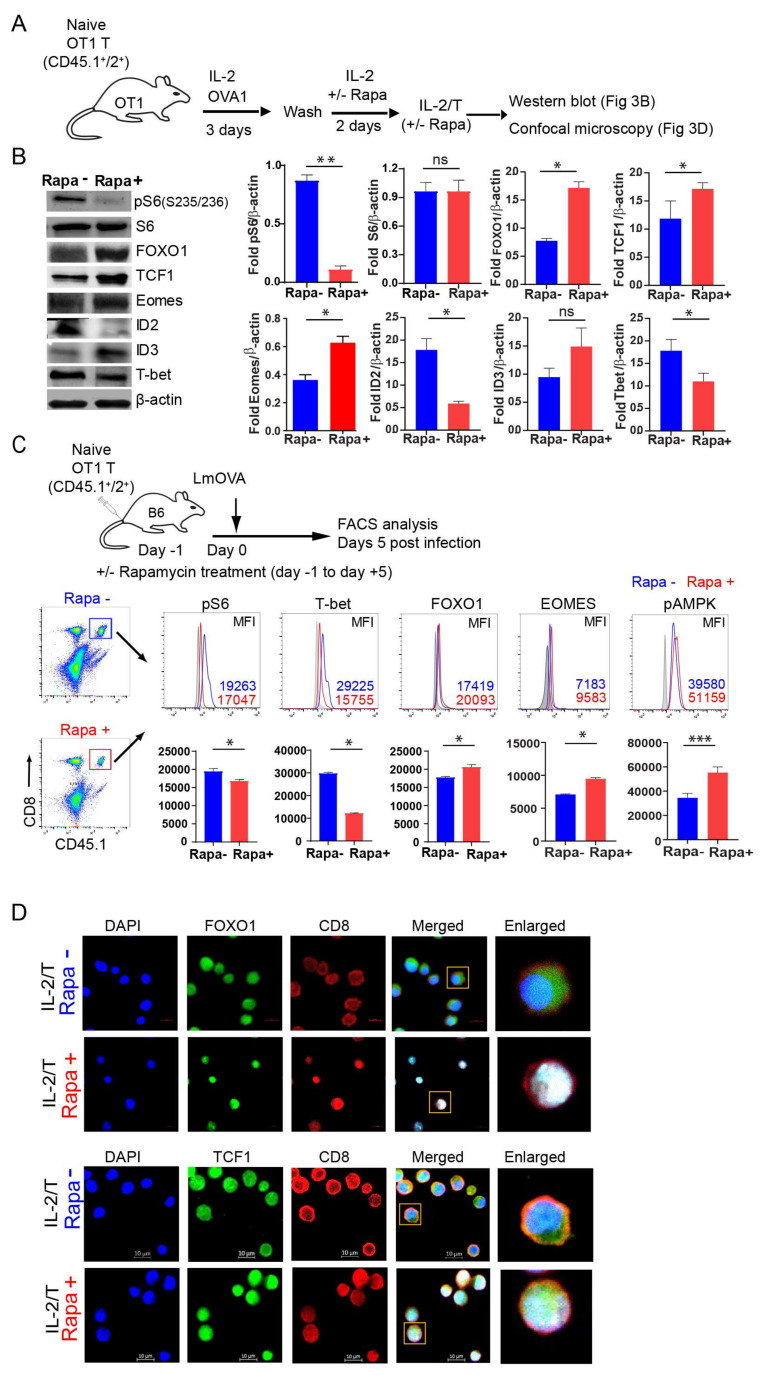
IL-2(Rapa+)/T-cells activate transcriptional FOXO1-TCF1-Eomes pathway. (**A**) Schematic diagram indicates splenic CD8^+^ T-cell culture using OT-1 mice (CD45.1^+^/2^+^) to generate rapamycin-treated or not treated T-cells. CD8^+^ T-cells were activated by 0.1 nM OVAI-peptide in IL-2 containing media for 3 days. Following the wash, activated T-cells were treated or not treated with rapamycin (100 nM) in IL-2 containing media for 2 additional days to form IL-2/T_E_ and IL-2(Rapa+)/T_M_ cells. (**B**) IL-2/T_E_ and IL-2(Rapa+)/T_M_ cells were used to prepare cell lysates for the Western blot analysis (left panels). Bar diagrams indicate relative fold change expression of various molecules compared to b-actin loading control. (**C**) Schematic diagram indicates adoptive transfer of naïve OVA-specific CD8^+^ T-cells (CD45.1^+^/2^+^) into C57BL/6 (CD45.2^+^) mice (*n* = 5), and 24 hr later infection (intravenous) with rLmOVA (2000 CFU/mice). Mice were treated with rapamycin from day -1 to day +5. Rapamycin not treated mice received saline and served as controls. Five days post-infection, adoptively transferred T-cells were tracked using CD45.1^+^ T-cells in total host CD8^+^ T-cells. The expression of signaling molecules and transcription factors was examined using intracellular staining by flow cytometry. Histograms (Figure 3C, upper panels) and bar diagrams (Figure 3C, lower panels) indicate relative expression of molecules in rapamycin-treated (red) and not treated (blue) groups. (**D**) Confocal microscopy for the cellular localization of FOXO1 (green) (Figure 3D, upper panels) and TCF1 (green) (Figure 3D, lower panels) in nuclear (blue) of IL-2/T_E_ and IL-2(Rapa+)/T_M_ cells. These T-cells were stained with PE-anti-CD8 antibody (red). Data presented as means ± SEM are representative of two independent experiments with similar results (*n* = 2–3/group/experiment). * *p* < 0.05, ** *p* < 0.01 by two-tailed Student’s *t*-test.

To further characterize the IL-2/T and IL-2(Rapa+)/T-cells, we performed Western blotting analyses to assess whether Rapa treatment inhibits mTORC1 activity in our experimental system (Figure 3A,B). We assessed the expression of ribosomal S6 which is routinely used as a proxy of mTORC1 activity [38]. We found Rapa treatment abolished the phosphorylated form of S6 (pS6, S_235/236_) in IL-2(Rapa+)/T, but not IL-2/T, cells (Figure 3B). Thus, our data indicate Rapa treatment inhibits mTORC1 activity. The transcription factors FOXO1, Id3, TCF1 and Eomes are known to regulate IL-7R^+^CD62L^+^KLRG1^−^ T_M_ cell differentiation, while Id2 and T-bet control IL-7R^−^CD62L^−^KLRG1^+^ T_E_ cell differentiation [39]. Among these factors, FOXO1 is upstream of TCF1 and Eomes and controls their activity [13,14,15,16,17], while Id3 is a downstream target of TCF1 that impinges upon T-cell memory [40]. We therefore measured the relative abundance of these proteins by Western blot analysis and found that IL-2(Rapa+)/T-cells displayed higher levels of FOXO1, TCF1, Eomes and Id3, but lower levels of Id2 and T-bet (Figure 3B). The reciprocal transcription factor expression profile was observed for IL-2/T-cells (Figure 3B). Therefore, our data indicate IL-2(Rapa+)/T-cells activate the FOXO1-TCF1-Eomes transcriptional pathway for T_M_ cell differentiation, while IL-2/T-cells trigger the T-bet transcriptional pathway for T_E_ cell differentiation. To further confirm that the IL-2(Rapa+)/T-cells prepared in vitro indeed activate the FOXO1-TCF1-Eomes transcriptional pathway, we adoptively transferred CD45.1^+^/2^+^ OTI naïve CD8^+^ T-cells into CD45.2^+^ B6 mice (to increase the frequencies of OTI T-cells) and then infected them with rLmOVA in the presence or absence of Rapa treatment (Figure 3C), as described in Figure 1A. The intracellular expression of transcription factors or kinases was determined through flow cytometry by gating on the CD8 and CD45.1 double-positive T-cell population (Figure 3C). We found that transferred OTI T-cells in Rapa-treated mice expressed higher levels of the transcription factors FOXO1 and Eomes, but lower levels of the pS6 (S_235/236_) kinase and transcription factor T-bet compared to untreated mice (Figure 3C), indicating Rapa also activates the T-cell transcriptional FOXO1 pathway in vivo. Active FOXO1 localizes to the nucleus while its phosphorylated form (pFOXO1) is inactive, and re-localizes to the cytosol where it is subsequently poly-ubiquitinated and degraded [17,41]. TCF1, a downstream target of Wnt signaling, plays a regulatory role in T-cell memory through its nuclear localization [17]. To visualize the subcellular localization of FOXO1 and TCF1, we performed confocal microscopy and found greater nuclear enrichment for both factors in IL-2(Rapa+)/T-cells compared to IL-2/T-cells (Figure 3D).

### 2.4. IL-2(Rapa+)/T-cells Activate the AMPKα1-ULK1-ATG7 Metabolic Axis

**Figure 4:**
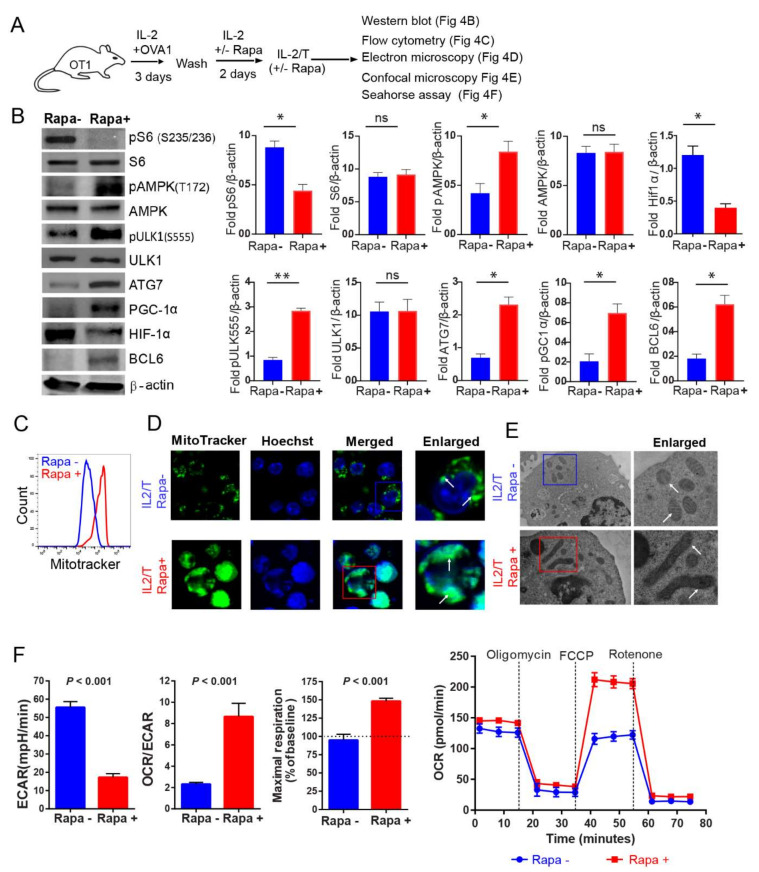
IL-2(Rapa+)/T-cells activate metabolic AMPK-ULK1-ATG7 pathway, promote mitochondrial biogenesis and induce FAO metabolism. (**A**) Schematic diagram indicates splenic CD8^+^ T-cell culture for generating rapamycin-treated or not treated T-cells. CD8^+^ T-cells were activated by 0.1 nM OVAI-peptide in IL-2 containing media for 3 days. Following the wash, activated T-cells were treated or not treated with rapamycin (100 nM) in IL-2 containing media for 2 additional days to form IL-2/T_E_ and IL-2(Rapa+)/T_M_ cells. (**B**) The IL-2/T_E_ and IL-2(Rapa+)/T_M_ cell lysates were examined by the Western blot analysis (left panels). Bar diagrams (right panels) indicate relative fold expression of various molecules compared to b-actin loading control. (**C**) Rapamycin treated (red) or not treated (blue) T-cells were stained with MitoTracker green fluorescent stain and analyzed by flow cytometry to measure the mitochondrial mass. (**D**) Confocal microscopy shows mitochondrial mass as indicated by MitoTracker green staining in rapamycin-treated or not treated T-cells. MitoTracker green stained mitochondria (white arrows) and Hoechst (blue for nucleus). (**E**) Electron microscopy images show large tubular and small round mitochondria (white arrows) in rapamycin-treated and not treated T-cells, respectively. (**F**) Bar diagrams show basal extracellular acidification rates (ECAR, left bar diagram panel), basal ECAR/OCR ratio (middle bar diagram panel), and maximal oxygen consumption rates (right bar diagram panel; horizontal dotted line indicates a basal OCR, and OCR values above this line is a mitochondrial spare respiratory capacity (SRC) in respective groups; Line diagram shows OCR measured in real-time under basal conditions and in response to indicated mitochondrial inhibitors (red—rapamycin-treated; blue—not treated with rapamycin); *p* < 0.0001 after FCCP injection. Data from two to three independent experiments are presented as means ± SEM (*n* = 4/group). * *p* < 0.05, ** *p* < 0.01, *** *p* < 0.001 by two-tailed Student’s *t*-test.

AMPKα1 is an evolutionarily conserved energy-sensing kinase that regulates catabolic processes by activating the autophagy-related molecules ULK1 and ATG7 [28] and stimulating mitochondrial biogenesis to drive the FAO required for T-cell memory [28]. The transcription factor HIF-1α is a master regulator of glycolytic gene expression and stimulates flux through this pathway [42]. In contrast, the repressor Bcl-6 down-regulates glycolytic flux [43]. We therefore assessed the relative abundance of pAMPKα1 (T_172_), pULK1 (S_555_), ATG7, Bcl-6 and HIF-1α in IL-2/T and IL-2(Rapa+)/T-cells by Western blot analysis (Figure 4A). We demonstrated that IL-2(Rapa+)/T-cells have higher levels of pAMPKα1 (T_172_), pULK1 (S_555_) and ATG7 (Figure 4B), suggesting that activation of the AMPKα1-ULK1-ATG7 metabolic axis promotes FAO and underlies the switch in fuel preference. Conversely, IL-2/T-cells exhibit the reciprocal expression profile for these factors and instead harbor more of the transcription factor HIF-1α, a master regulator for glycolysis. We also measured the relative abundance of pAMPKα1 (T_172_) in OTI T-cells and found that those isolated from Rapa-treated mice had higher levels of pAMPKα1 (T_172_) (Figure 3C). This finding indicates that Rapa also activates the AMPKα1 metabolic pathway in T-cells in vivo.

### 2.5. IL-2(Rapa+)/T-cells Promote Mitochondrial Biogenesis

AMPK-peroxisome proliferator-activated receptor-gamma coactivator-1α (PGC-1α) is a critical regulator of mitochondrial biogenesis [44]. To assess whether enhanced activity of the AMPKα1-ULK1-ATG7 metabolic axis affects the expression of PGC-1α to modulate organelle content, we performed Western blot analysis. Indeed, we found IL-2(Rapa+)/T-cells displayed higher levels of PGC-1α compared to IL-2/T-cells (Figure 4B). To further confirm this finding, we performed flow cytometry and confocal microscopy analyses using MitoTracker Green, which binds to the mitochondrial membranes and stains the organelle [45]. We demonstrated that IL-2(Rapa+)/T-cells contain a fragmented reticulum with many more individual mitochondria than IL-2/T-cells (Figure 4C,D). Mitochondrial morphology has previously been shown to affect fuel preference in T-cells, with small, round organelles supporting glycolysis in T_E_ cells and long, tubular mitochondria being central to FAO in T_M_ cells [46]. Consistent with this idea, our electron microscopy analysis showed that mitochondria were elongated and tubular in IL-2(Rapa+)/T-cells while they were small and spherical in IL-2/T-cells (Figure 4E).

### 2.6. IL-2(Rapa+)/T-cells Have Substantial Mitochondrial SRC and Rely on FAO

Mitochondria are bioenergetic organelles controlling energy homeostasis that contribute to T_M_ cell survival [47] via OXPHOS, by providing the spare respiratory capacity (SRC) essential for FAO [48,49]. Therefore, to assess the effect of Rapa treatment on mitochondrial metabolism in T-cells, we measured the bioenergetic profiles of IL-2/T and IL-2(Rapa+)/T-cells under basal conditions and following the addition of various agents to block flux through the electron transport chain (ETC) or impair ATP synthesis. We demonstrated that IL-2(Rapa+)/T-cells produced more ATP than IL-2/T-cells (Figure 4F). Moreover, IL-2(Rapa+)/T-cells had a higher rate of O_2_ consumption (OCR) when compared to the rate of extracellular acidification (ECAR) (Figure 4F), which is a marker of FAO and indicates preferential usage of fatty acids over sugars as a fuel source. In contrast, IL-2/T-cells had a lower OCR and an elevated ECAR, indicating they relied more on glycolytic flux to maintain energy homeostasis compared to IL-2(Rapa+)/T-cells (Figure 4F). Finally, the maximal OCR following FCCP injection was significantly higher in IL-2(Rapa+)/T-cells, consistent with the idea that the SRC allows these cells to produce more ATP via OXPHOS than IL-2/T-cells and utilize FAO metabolism to preserve energy homeostasis.

### 2.7. AMPKα1 Deficiency in IL-2(Rapa+)/T-cells Reduces Mitochondrial Biogenesis, but Up-Regulates HIF-1α Expression and Induces a Metabolic Switch from FAO to Glycolysis

**Figure 5:**
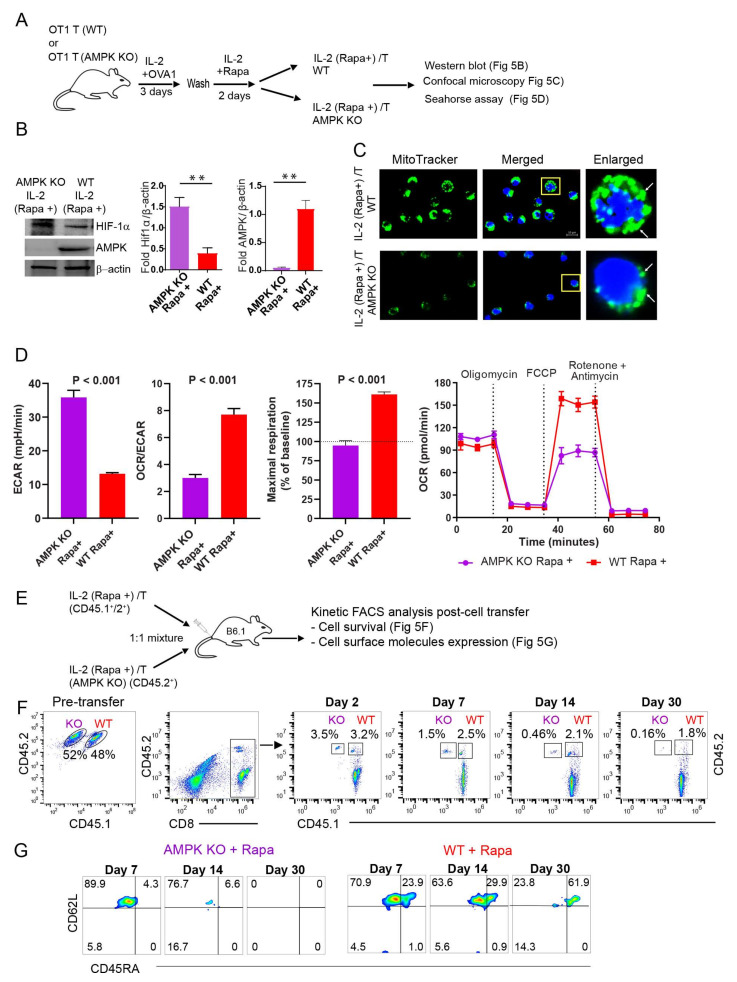
AMPKα1 deficiency abolishes metabolic AMPKα1 pathway, reduces mitochondrial biogenesis and induces a metabolic switch from FAO to glycolysis in AMPKα1 KO IL-2(Rapa+)/T-cells. (**A**) Schematic diagram indicates splenic CD8^+^ T-cell culture for generating rapamycin-treated T-cells from wild-type (WT) and AMPKα1 KO. OVA-specific naïve CD8^+^ T-cells from the WT OT-1 mice or from AMPKα1 KO mice were activated by 0.1 nM OVAI-peptide in IL-2 containing media for 3 days. Following the wash, activated T-cells were treated with rapamycin (100 nM) in IL-2 containing media for 2 additional days to form IL-2(Rapa+)/T_M_ and AMPK KO IL-2(Rapa+)/T_M_ cells. (**B**) Cell lysates from IL-2(Rapa+)/T_M_ and AMPK KO IL-2(Rapa+)/T_M_ cells were examined by the Western blot analysis. (**C**) Confocal microscopy shows mitochondrial mass as indicated by MitoTracker green staining in IL-2(Rapa+)/T_M_ and AMPK KO IL-2(Rapa+)/T_M_ cells. MitoTracker green stained mitochondria (white arrow) and Hoechst stained nucleus (blue). (**D**) Bar diagrams show basal extracellular acidification rates (ECAR, left bar diagram panel), basal ECAR/OCR ratio (middle bar diagram panel), and maximal oxygen consumption rates (right bar diagram panel; horizontal dotted line indicates a basal OCR, and OCR values above this line is a mitochondrial spare respiratory capacity (SRC) in respective groups; Line diagram shows OCR measured in real-time under basal conditions and in response to indicated mitochondrial inhibitors (red—WT; purple—AMPKα1 KO); *p* < 0.0001 after FCCP injection. (**E**) The schematic diagram indicates rapamycin-treated T-cells generated from congenic OT-1 mice using WT mice from CD45.1^+^/2^+^ background and AMPKα1 KO from CD45.2^+^ background. Rapamycin treated wild type (CD45.1^+^/2^+^) or rapamycin-treated AMPKα1 KO (CD45.2^+^) effector T-cells were mixed 1:1 and adoptively transferred into CD45.1 mice. (**F**) The cell survival kinetics of adoptively transferred WT (CD45.1^+^/2^+^) or AMPKα1 KO (CD45.2^+^) effector T-cells in total host CD8^+^ T-cells were analyzed longitudinally in peripheral blood by flow cytometry. Flow panels show the relative percentage of rapamycin-treated WT (CD45.1^+^/2^+^) or AMPKα1 KO (CD45.2^+^) effector T-cells post-adoptive transfer (*n* = 4). (**G**) Flow panels indicate cell phenotype of rapamycin-treated WT (CD45.1^+^/2^+^) or rapamycin-treated AMPKα1 KO (CD45.2^+^) T-cells at indicated days post-adoptive transfer (*n* = 4). Data shown are from two to three independent experiments and are presented as means ± SEM (*n* = 4/group). * *p* < 0.05, ** *p* < 0.01, *** *p* < 0.001 by two-tailed Student’s *t*-test.

To confirm a critical regulatory role for AMPKα1 in the reliance of IL-2(Rapa+)/T-cells on FAO for their metabolic fitness, we repeated the above experiments using IL-2(Rapa+)/T and AMPKα1 KO IL-2(Rapa+)/T-cells derived from CD45.1^+^/45.2^+^ WT OTI and CD45.2^+^ AMPKα1 KO/OTI mice, respectively (Figure 5A). First, we confirmed that AMPKα1 was undetectable by Western blot in AMPKα1 KO IL-2(Rapa+)/T-cells (Figure 5B). Next, we demonstrated that AMPKα1 KO IL-2(Rapa+)/T-cells also had less mitochondrial mass (Figure 5C) and lower rates of OXPHOS metabolism using the OCR/ECAR ratio as a proxy (Figure 5D). However, AMPKα1 KO IL-2(Rapa+)/T-cells expressed more of the transcription factor HIF-1α (Figure 5B), and had an increased ECAR, indicative of enhanced glycolytic flux (Figure 5D). Interestingly, genetic ablation of AMPKα1 in IL-2(Rapa+)/T-cells eliminated their SRC (Figure 5D). These data collectively suggest that the loss of AMPKα1 in IL-2(Rapa+)/T-cells leads to activation of HIF-1α and a switch in fuel preference from FAO to glycolysis.

### 2.8. AMPKα1 Deficiency Down-Regulates CD45RA Expression in IL-2(Rapa+)/T-cells and Abolishes Their Long-Term Survival

To measure cell survival, an equal number of CD45.1^+^/2^+^ IL-2(Rapa+)/T and AMPKα1 KO CD45.2^+^ IL-2(Rapa+)/T-cells prepared in vitro were adoptively transferred into CD45.1^+^ B6.1 mice, and cell survival and expression of the T_SCM_ marker CD45RA were kinetically monitored by flow cytometry (Figure 5E), as described in Figure 2B. These analyses demonstrated that the survival IL-2(Rapa+)/T-cells (1.8%) was more than 10-fold higher than that of AMPKα1 KO IL-2(Rapa+)/T-cells 30 days post-cell transfer (Figure 5F), indicating AMPKα1 is required for the ability of Rapa to promoter long-term survival of T_M_ cells. In addition, AMPKα1 deficient IL-2(Rapa+)/T-cells were almost devoid of CD45RA expression even at 7 days post-cell transfer (Figure 5G), indicating the kinase is essential to sustain expression of this cell surface molecule.

## 3. Discussion

Rapa inhibits the kinase activity of mTOR by forming complexes with its intracellular receptor FK506-binding protein-12 (FKBP12), which then bind to the N-terminal domain of mTOR and interfere with mTORC1 and mTORC2 assemblies [50]. Depending on the nature of the Rapa exposure, it can uniquely inhibit mTORC1 and mTORC2 formations owing to their differential sensitivities. For example, low Rapa concentrations or transitional exposure to Rapa only inhibit mTORC1 assembly, while high Rapa concentrations or long-term exposure to Rapa are capable of limiting mTORC2 formation [50].

**Figure 6:**
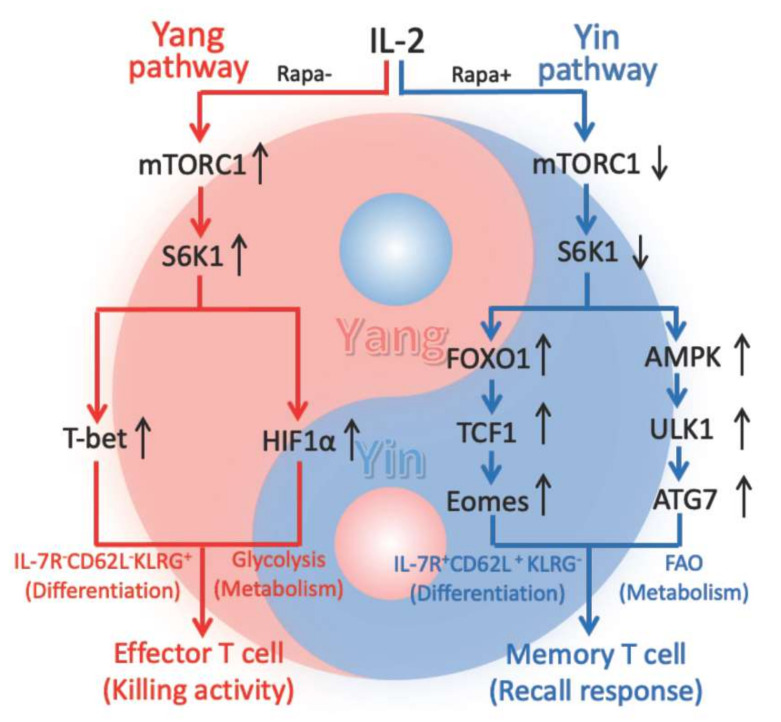
Schematic diagram showing that IL-2(Rapa+)/T with weak mTORC1/S6K (weak yang) but strong AMPK (yin) signaling become T-cell memory via activation of the (yin) FOXO1-TCF1-Eomes transcriptional pathway and the (yin) AMPK-ULK1-ATG7 metabolic axis, whereas IL-2/T-cells with strong mTOC1/S6K1 (yang) signaling differentiate into effector T-cells via activation of the (yang) T-bet transcriptional and the (yang) HIF-1α metabolic pathways. In the figure, red color part represents yang controlling cell growth, differentiation and functional effect, and using glycolysis for cell metabolic energy while blue color part represents yin regulating cell quiescence, and using FAO for cell metabolic energy.

Rapa-inhibition of mTORC1 (RIM) promoted CD8^+^ T-cell memory [21,22,23,24]. However, the transcriptional and metabolic pathways by which RIM promotes long-term CD8^+^ T-cell memory are not well elucidated. In this study, we demonstrate that Rapa promotes T-cell memory in vivo post-infection of mice with *Listeria monocytogenesis* rLmOVA and stimulates the transition of effector T (T_E_) to memory T (T_M_) cells in vitro. IL-2- and IL-2+Rapa-stimulated T [IL-2/T and IL-2(Rapa+)/T] cells with high and low levels of mTORC1-S6K signaling differentiate into short-term IL-7R^−^CD62L^−^KLRG1^+^ T_E_ and long-lived IL-7R^+^CD62L^+^KLRG1^−^ T_M_ cells, respectively. To assess the underlying pathways, we performed Western blotting, confocal microscopy and Seahorse-assay analyses using IL-2/T_E_ and IL-2(Rapa+)/T_M_ cells. We determined that IL-2(Rapa+)/T_M_ cells activate the FOXO1, TCF1 and Eomes transcription factors and the metabolic regulators pAMPKα1(T_172_), pULK1(S_555_) and ATG7 to promote mitochondrial biogenesis and FAO, while down-regulating the transcription factors T-bet and HIF-1α. We observed the reciprocal expression pattern for these molecules in IL-2/T_E_ cells. Therefore, our data indicate that Rapa-inhibition of mTORC1-S6K promotes T-cell memory via activation of the FOXO1-TCF1-Eomes transcriptional pathway and the AMPKα1-ULK1-ATG7 metabolic axis, whereas IL-2-stimulated strong mTORC1-S6K signaling controls T_E_ cell formation via activation of the transcriptional T-bet and metabolic HIF-1α pathways (Figure 6).

It has been demonstrated that the FOXO1-TCF1-Eomes transcriptional pathway is indispensable for T_M_ cell differentiation and formation [17]. In this study, we used IL-2(Rapa+)/T-cells derived from AMPKα1 KO/OTI mice to demonstrate that AMPKα1 deficiency ablates the AMPKα1 pathway, blunts mitochondrial biogenesis, induces lower rates of OXPHOS metabolism and abolishes long-term T-cell survival, indicating that the AMPKα1-ULK1-ATG7 metabolic axis is also indispensable for CD8^+^ T_M_ cell formation. Therefore, we conclude that RIM promotes T-cell memory by coordinate regulation of the FOXO1-TCF1-Eomes transcriptional program and the AMPKα1-ULK1-ATG7 metabolic axis (Figure 6).

The CD8^+^ T_M_ cell population was originally divided into two subsets: CCR7^−^CD62L^−^IL-7R^+^ effector memory T (T_EM_) and CCR7^+^CD62L^+^IL-7R^+^ central memory T (T_CM_) cells [10,11]. Due to the lymph node homing receptors CCR7 and CD62L, CCR7^+^CD62L^+^IL-7R^+^ T_CM_ cells mainly reside in lymph nodes while CCR7^−^CD62L^−^IL-7R^+^ T_EM_ cells are mostly localized to peripheral tissues [10,11]. Recently, a new subset of the T_M_ cell population known as CD45RA^+^IL-7R^+^CD62L^+^ T_SCM_ cells has been defined, and shows increased proliferative activity, superior antitumor immunity, and the capacity to differentiate into T_EM_ and T_CM_ cells [32]. These T_SCM_-cells express two naïve T-cell markers CD45RA and IL-7R [32], which are critical for T-cell differentiation and homeostasis as well as for the regulation of T-cell signaling threshold [12,51,52]. Up-regulation of IL-7R expression in T_M_ cells is directly regulated by the transcription factor FOXO1 [53]. However, except for a few reports, molecular regulation of T-cell CD45RA expression is less well studied. It was reported that IL-7 drives the phenotypic revision of IL-7R^+^CD62L^+^ T_CM_ and IL-7R^+^CD62L^−^ T_EM_ cells into CD45RA^+^IL-7R^+^CD62L^+^ T_SCM_ cells by facilitating the epigenetic reorganization essential for secondary responses [54]. Metabolic control of T-cell immunity via its intermediary metabolites is a newly emerging area of fundamental immunology [55]. In this study, we demonstrate that Rapa treatment promotes the transition of IL-2/T_E_ cells into IL-2(Rapa+)/T_M_ cells in vitro. Interestingly, we also observe that AMPKα1 deficiency in IL-2(Rapa+)/T-cells leads to the down-regulation of CD45RA, but not IL-7R, expression. This finding suggests a potential association between AMPKα1-regulated FAO metabolism and CD45RA expression, and indicates Rapa-promoted CD45RA^+^IL-7R^+^CD62L^+^ T_SCM_ differentiation is mediated by AMPKα1 activity. Therefore, the underlying mechanism warrants further study.

mTORC1 and AMPKα1 are two evolutionally conserved signaling molecules that regulate cell metabolism and differentiation [18,19]. However, their interplay in controlling CD8^+^ T-cell differentiation is less understood. In this study, we demonstrate that IL-2 stimulates strong mTORC1-S6K activity but very weak AMPKα1 signaling, leading to IL-2/T_E_ cell differentiation. However, when mTORC1 is inhibited by Rapa, IL-2+Rapa stimulation induces IL-2(Rapa+)/T_M_ cell formation via activation of the metabolic AMPKα1 pathway. This finding argues that mTORC1 suppresses AMPKα1 in IL-2/T_E_ cells, perhaps via Raptor-dependent phosphorylation of AMPKα1 at S_347_ to block its phosphorylation at T_172_ and inhibit its subsequent activation [56]. However, when AMPKα1 is absent, IL-2(Rapa+)/T-cells derived from AMPKα1 KO/OTI mice lose T_M_ cell formation and long-term T-cell survival. Instead, the mTORC1-S6K-controled transcription factor HIF-1α [42] was up-regulated in IL-2(Rapa+)/T-cells lacking AMPKα1, which leads to a metabolic switch from FAO to glycolysis. This finding indicates that AMPKα1 normally inhibits mTORC1 in IL-2(Rapa+)/T-cells and is supported by earlier studies which showed that AMPKα1 activators such as 5-aminoimidazole-4-carboxamide-1-β-4-ribofuranoside (AICAR) and metformin enhance the inhibitory effect of Rapa on mTORC1 [57,58]. The inhibitory effect of AMPKα1 on mTORC1 may be due to its ability to phosphorylate the mTORC1 binding partner Raptor at S_722_ and S_792_ [59] or to activate the mTORC1 suppressor TSC2 [38,60,61]. Therefore, our data collectively indicate a negative feedback mechanism exists between these two energy sensors in CD8^+^ T-cell differentiation and suggest that their relative activities play an important role in immune responses and immune diseases [2,62].

The theory of yin (negative regulation) and yang (positive regulation) with a negative feedback interplay represents one of the most fundamental principles in traditional Chinese medicine. This theory has been applied to interpret the immune system. For example, CD28 and CTLA-4 have been described as the yin and yang of T-cell co-stimulation [63]. The yin and yang interplay of cytokine IFN-γ in inflammation and autoimmune diseases has also been reported [64]. It has also been suggested that Th1 cells belong to yang whereas Th2 and Treg cells belong to yin, where yang represents immune initiation and response while yin represents immune regulation and tolerance [65]. Recently, it was reported that AMPK represents the yin that signals a lack of nutrients and inhibits cell growth, whereas mTORC1 represents the yang that signals the availability of nutrients and promotes cell growth [66]. However, how this theory informs immune responses at the molecular level is still unknown. Our study is significant in this regard for it presents the first evidence showing how mTORC1 acts as the yang and AMPK acts as the yin in generating signals that control CD8^+^ T-cell differentiation into effector and memory T-cells (Figure 6). Specifically, (i) IL-2-stimulated strong mTORC1 (yang) signaling in IL-2/T-cells controls cell expansion with glycolysis metabolism for cell energy and differentiation into IL-2/T_E_ cells with effector function via activation of transcriptional T-bet and metabolic HIF-1α (yang) pathways; (ii) IL-2+Rapa-stimulation results in weak mTORC1 (yang) but strong AMPKα1 (yin) signaling and regulates cell differentiation into IL-2(Rapa+)/T_M_ cells that are quiescent and uses FAO metabolism for cell energy (Figure 6), and (iii) AMPKα1 (yin) and mTORC1 (yang) interplay under a negative feedback mechanism in CD8^+^ T-cell differentiation.

Inhibition of mTORC1 can alter the intrinsic properties of CD8^+^ T-cells to favor their differentiation into memory T-cells and, as such, may be an attractive strategy to enhance therapeutic anti-tumor immunity. Therefore, elucidation of the exact molecular signaling through mTORC1 that controls the transcriptional FOXO1 and metabolic AMPKα1 pathways for T-cell memory may open up new avenues for developing new therapeutics to enhance T_M_ cell responses in humans. An additional therapeutic target of interest in this context is phospholipase D (PLD), an enzyme that catalyzes the hydrolysis of the major phospholipid phosphatidylcholine within cell membrane [67]. It has been reported that the phosphatidic acid (PA) produced by PLD plays an important role in T-cell receptor-mediated activation via its activation and stabilization of mTORC1 and mTORC2 [50,67]. Therefore, we speculate that inhibition of PLD may also promote T-cell memory through down-regulation of mTOC1 and mTORC2 [17,21].

The ultimate goal of vaccine development is to stimulate hosts to produce a large number of good quality T_M_ cells against cancer and infectious diseases [3]. This goal will be greatly impacted by our new finding that Rapa promotes CD8^+^ T-cell memory via the coupled regulation of the FOXO1-TCF1-Eomes transcriptional pathway and the AMPKα1-ULK1-ATG7 metabolic axis (Figure 6). Accumulating evidence highlights the integration of transcriptional and metabolic pathways, with transcription factors shaping metabolic programming of T-cell memory and cellular metabolites reciprocally regulating the expression of genes involved in T-cell memory programming at the epigenetic and transcriptional levels [8,68]. Therefore, ongoing investigation of new metabolic or epigenetic signals critical to T-cell memory programs is warranted and will greatly advance mTORC1-targeted immunotherapeutics and vaccine development for cancer and infectious diseases.

Taken together, we provide the first evidence that RIM promotes CD8^+^ T-cell memory by coupling the FOXO1-TCF1-Eomes transcriptional pathway and the AMPKα1-ULK1-ATG7 metabolic axis and that the energy sensor AMPKα1 plays a critical role in RIM-induced CD8^+^ T-cell memory. Our findings collectively provide novel mechanistic insight into how inhibition of the mTORC1 pathway impinges upon CD8^+^ T-cell memory that will greatly impact vaccine development and immunotherapy for cancer and infectious diseases.

## 4. Materials and Methods

### 4.1. Experimental Animals

Female wild-type (WT) C57BL/6 mice (CD45.2^+^, #000664), CD45.1^+^ B6.1 SJL-PtprcaPepcb/BoyJ mice (#2014), OVAI peptide (OVA_257–264_)-specific TCR (T-cell receptor) transgenic OT-I (C57BL/6-Tg (TcraTcrb)1100Mjb/J) (OT-I/CD45.2^+^, #003831) mice, C57BL/6-AMPKα1 flox/flox mice (#014141), and CD4-Cre mice (#0022071) were purchased from the Jackson Laboratory (Bar Harbor, MA, USA). B6.1 OT-I (CD45.1^+^/45.2^+^) mice were obtained by breeding B6/OT-I(CD45.2^+^) mice with B6.1 (CD45.1^+^) mice. AMPKα1 KO OT1 mice were generated by first crossing AMPKα1 flox/flox mice with CD4-Cre mice to obtain T-cell specific AMPKα1 KO (T-AMPKα1 KO) mice, which were further crossed with OT1 mice to generate T-AMPKα1 KO OT1 mice. Mice were kept under specific pathogen-free conditions in the animal facility at the Health Sciences Building. All mice were maintained according to the protocols approved by Animal Use and Care Committee of the University of Saskatchewan. All experiments were repeated at least two times and included four to six mice per group. All experiments were conducted according to protocols and guidelines approved by the Animal Research Ethics Board, University of Saskatchewan (protocol # 20180065).

### 4.2. Rapamycin Treatment in Mice Challenged with rLmOVA

rLmOVA challenge (2500 colony-forming unit) was performed with a published strain by intravenous injection in the tail vein [21,69]. C57BL/6 mice were injected (intraperitoneal) daily with rapamycin (75 μg /kg body-weight) (Sigma, Markham, OT, Canada) during the T-cell expansion phase (day -1 before rLmOVA infection until day +7 post-infection) [21]. On days 7, 15, 30, and 60 after LmOVA infection, OVA-specific CTL responses were analyzed in blood samples by flow cytometry using PE-H-2K^b^/OVA_257–264_ tetramer (MBL LTD) and FITC-anti-CD8 antibody (BD Biosciences).

### 4.3. Splenocytes and Peripheral Blood Mononuclear Cell Preparation

Single-cell suspensions from spleens were prepared by mashing up the organs with a 10-mL syringe plunger against a 70-μm cell strainer into 10-mL RPMI 1640 medium (RPMI) supplemented with 10% (*v*/*v*) fetal calf serum (FCS) and 20 μg/mL gentamicin. Peripheral blood samples were collected into tubes containing heparin (BD Biosciences, San Jose, CA, USA) by nicking the lateral tail vein. Red blood cells in splenocytes and peripheral blood were lysed with ammonium-chloride-potassium (ACK) lysing buffer (150 mM NH4Cl, 10 mM KHCO3, and 0.1 mM EDTA) by incubating for 5-min at room temperature, followed by quenching with RPMI medium, centrifugation, and re-suspension in 5 mL of RPMI medium.

### 4.4. Preparation of In Vitro Activated T-cells

Naïve CD8^+^ T-cells derived from CD45.1^+^/2^+^ B6.1/OT-I and CD45.2^+^ OT-I or AMPKα1 KO/OTI mouse splenocytes were isolated using EasySep™ CD8^+^ T-cell Purification Kits (Stem Cells Tech, Vancouver, BC, Canada) according to the manufacturer’s protocols. Purified CD8^+^ T were cultured in 24-well plates (1 × 10^6^ cells/well) in 2 mL of Complete RPMI medium (CM) containing 10% FCS, and 50 mM 2-mercaptoethanol and activated with 0.1 nM OVAI peptide (OVA_257–264_, SIINFEKL) in the presence of IL-2 (100 U/ mL) for 3 d, followed by another 2 d of culturing in IL-2 (100 U/mL) without Rapa for CD45.1^+^/2^+^ IL-2/T_E_ cells and culturing in IL-2 (100 U/mL) with Rapa (100 nM) for CD45.2^+^ IL-2(Rapa+)/T_M_, CD45.1^+^/2^+^ IL-2(Rapa+)/T_M_ and CD45.2^+^ AMPKα1 KO IL-2(Rapa+)/T_M_ cells [21].

### 4.5. Adoptive T-cell Transfer and Kinetic Flow Cytometry Analyses

The above in vitro prepared CD45.1^+^/2^+^ IL-2/T_E_ and CD45.2^+^ IL-2(Rapa^+^)/T_M_ cells were mixed at a 1:1 ratio, and then 1 × 10^7^ mixed T-cells were injected i.v. into CD45.1^+^ B6.1 host mice. In another experiment, the above in vitro prepared CD45.1^+^/2^+^ IL-2(Rapa+)/T_M_ and CD45.2^+^ AMPKα1 KO IL-2(Rapa^+^)/T_M_ cells were mixed at a 1:1 ratio, and then 1 × 10^7^ mixed T-cells were injected i.v. into CD45.1^+^ B6.1 host mice. T-cell survival was kinetically measured using mouse peripheral blood samples stained with FITC-labelled anti-CD45.1 and PE-labelled anti-CD45.2 antibodies by flow cytometry analysis. Using this method, adoptively transferred CD45.1^+^/2^+^ IL-2/T and CD45.2^+^ IL-2(Rapa^+^)/T-cells or CD45.1^+^/2^+^ IL-2(Rapa+)/T_M_ and CD45.2^+^ AMPKα1 KO IL-2(Rapa^+^)/T_M_ cells were separately detected for further measurement of T-cell memory markers CD62L and CD45RA.

### 4.6. Flow Cytometry

Surface staining was performed by incubating the cells with fluorescently labeled antibodies for 30 min on ice in PBS supplemented with 2% FCS and 0.1% sodium azide. The following antibodies used for cell surface staining were purchased from Biolegend (San Diego, CA, USA): PE594 Cy5-CD8 (clone53-6.7), FITC-CD45.1 (clone A20), Alexa Fluor 700-CD45.2 (clone 104), APC KLRG1 (clone 2F1), Brilliant Violet 510-IL-7Ra (clone A7R34), and APC-A750-CD62L (clone MEL-14). Brilliant Violet 421-CD45RA (clone 14.8) antibody was obtained from BD Biosciences. Intracellular staining was done as per the manufacturer’s (BD Bioscience) protocol for flow cytometry. Briefly, after cell-surface staining, T-cells were fixed and permeabilized in 1 mL of fixation and permeabilization working solution for 20 min on ice, followed by a wash in 1X permeabilization solution and incubation with primary antibodies against T-bet, FOXO1, and pS6 (S_235/236_) (Cell Signaling, Danvers, MA, USA) in 100 μL of 1X permeabilization buffer. Cells were washed once with 1 mL of 1X permeabilization solution, followed by incubation with PE-goat anti-rabbit IgG secondary antibody (Biolegend) in 100 μL of 1X permeabilization buffer. The samples were then washed twice with 1X permeabilization solution and re-suspended in PBS supplemented with 2% FCS and 0.1% sodium azide for flow cytometry analyses. The cell mitochondrial mass was measured by staining cells with MitoTracker Green (Life Technologies, Carlsbad, CA, USA). Briefly, cells were stained with MitoTracker Green at 10 nM for 15 min at 37°C according to the manufacturer’s manual. The cells were washed three times with PBS and then analyzed by flow cytometry, as we previously described [45]. Flow cytometry data were acquired using CytoFLEX (Beckman Coulter, Brea, CA, USA) and analyzed with FlowJo software (TreeStar, Ashland, OR, USA).

### 4.7. Confocal and Electron Microscopy Imaging

Mito Tracker Green (Life Technologies) was used to measure mitochondrial mass. In vitro Rapa-treated (Rapa+) and untreated (Rapa−) T-cells were stained with 50 nM MitoTracker Green or 10 μM TMRM for 15 min at 37 °C, according to the manufacturer’s manual. Stained cells were washed three times with PBS, followed by incubation with 5 μg/mL of Hoechst 33342 solution (Life Technologies) for 20 min at room temperature. Confocal images were acquired using the Zeiss LSM700 confocal microscope (Carl Zeiss, Oberkochen, BW, Germany) with ×20 objective [45]. For electron microscope imaging, cell pellets (2 × 10^6^ T-cells/each) were fixed in 2% paraformaldehyde and 2.5% glutaraldehyde in 100 mM sodium cocodylate. After fixation, samples were washed in cacodylate buffer and fixed in 1% osmium tetroxide. After extensive washing in H_2_O, samples were stained with 1% aqueous uranyl acetate for 1 h and washed again. Samples were dehydrated in ethanol, embedded in Eponate 12™ resin (Ted Pella, Redding, CA, USA), and sectioned for imaging [70]. Images were acquired using a JOEL 1200 EX transmission electron microscope.

### 4.8. Immunoblotting

Cells were lysed in ice-cold RIPA lysing buffer (Thermo Scientific, MA, USA). Cell lysates were separated by SDS-PAGE and transferred to a nitrocellulose membrane. The membrane was blocked with 5% BSA in PBS and examined by immunoblot analysis using various antibodies. Blots were probed with antibodies recognizing AMPKα1, pAMPKα1 (T_172_), S6, pS6 (S_235/236_), ULK1, pULK1 (S_555_), ATG7, T-bet, PGC1α, FOXO1, TCF1, HIF1α, Id2, Id3, anti-prokaryotic translation initiation factor-4E (S_209_), anti-pS6 (S_235/236_), anti-EOMES, anti-T-bet, and anti-β-actin (Cell Signaling Technology, Danvers, MA, USA). The secondary antibodies horseradish peroxidase (HRP)-conjugated anti-mouse/anti-rabbit IgG (Cell Signaling) were used. The membrane was scanned under the Image Doc instrument according to the manufacturer’s instructions (Bio-rad Hercules, CA, USA) [44,69]. Band intensities were analyzed using Image J software.

### 4.9. Seahorse Assays

The OCR and ECAR of in vitro-activated CD8^+^ T-cells were measured in XF RPMI media containing 25 mM glucose, 2 mM L-glutamine, and 1 mM sodium pyruvate (Agilent, Lexington, MA, USA). Cells were then plated onto XF8 cell culture microplates (1.5 × 10^5^ cells per well) coated with poly-D-lysine (Sigma-Aldrich) to facilitate T-cell attachment. A mitochondrial stress test was performed by measuring OCR (pmol min-1) at the basal level and after sequential injection of oligomycin (1.5 μM), FCCP (2.5 μM), and rotenone/antimycin A (0.5 μM) (Agilent, CA, USA), and run on an Agilent Seahorse XFp analyzer (Seahorse Bioscience, Agilent, CA, USA). The following assay conditions were used for the experiments with the Seahorse system: 3 min mixture; 0 min wait; and 3 min measurement.

### 4.10. Statistical Analysis

Statistical analyses were performed using unpaired *t*-test (two-tailed) or analysis of variance for comparison of means using GraphPad Prism6 software (GraphPad, La Jolla, CA, USA) [44,69]. Values of *p <* 0.05 and <0.01 were considered significant and very significant, respectively.

## Data Availability

Data are contained within the article.

## Abbreviations

AMPKα1	adenosine monophosphate-activated protein kinase-α1
ATG7	autophagy-related gene-7
ECAR	extracellular acidification rate
FAO	fatty acid oxidation
FOXO1	forkhead box protein-O1
KLRG1	killer cell lectin-like receptor subfamily G member-1
KO	knockout
MPEC	memory precursor effector cell
mTORC1	mammalian target of rapamycin complex-1
OCR	O2 consumption rate
OXPHOS	oxidative phosphorylation
Rapa	rapamycin
S6K1	S6 kinase
SLEC	short-lived effector cell
SRC	spare respiratory capacity
TCF1	T-cell factor-1
T_E_ cell	effector T-cell
T_M_ cell	memory T-cell
IL-2/T-cell	IL-2-stimulated T-cell
IL-2(Rapa+)/T-cell	IL-2+Rapa-stimulated T-cell
ULK1	Unc-51-like autophagy-activating kinase-1
WT	wild-type
